# Age-related and individual features of the HPA axis stress responsiveness under constant light in nonhuman primates

**DOI:** 10.3389/fendo.2022.1051882

**Published:** 2023-01-09

**Authors:** Nadezhda Goncharova, Olga Chigarova, Tamara Oganyan

**Affiliations:** Laboratory of Experimental Endocrinology, Research Institute of Medical Primatology, Sochi, Russia

**Keywords:** acute stress, the HPA axis, melatonin, constant light, aging, behavior, rhesus monkeys

## Abstract

The hypothalamic-pituitary-adrenal (HPA) axis is a key adaptive neuroendocrine system, dysfunction of which plays an important role in the increasing incidence of stress-dependent age-related pathology. Among the environmental factors effecting increase age-related diseases, great importance is given to disturbances of the light-dark schedule, particularly with increased illumination at night. While disruption of the light-dark schedule has long been recognized as a powerful behavioral stressor, little is known regarding stress reactivity of the HPA under constant light (CL) conditions, especially with aging and depending on the features of stress behavior. The purpose of this investigation was to study the age-related and individual features of the HPA axis response to acute stress exposure (ASE) under chronic CL in nonhuman primates that are known to differ in behavioral responsiveness to stress. Young and old female rhesus monkeys (with control standard behavior or anxiety and depression-like behavior) were exposed to CL (24 h light/day, 330-400 lux for 4 to 8 weeks). Control young and old monkeys were exposed to standard lighting (SL) with natural light during the day and darkness at night. All animals were subjected to ASE (restriction of mobility for 2 hours), functional tests with corticotrophin-releasing hormone and arginine-vasopressin, and study of circadian rhythms of cortisol and pineal melatonin secretion. For the first time an inhibitory effect of CL on the reaction of the adrenal cortex to ASE was revealed in all individuals, regardless of age and preexisting behavior stress reactivity, the mechanisms of which were age-dependent: due to inhibition of the pituitary ACTH secretion in young animals and mainly not affecting the ACTH secretion in old individuals. There were no significant changes in melatonin secretion both in young and old animals. The observed CL inhibition of adrenal cortical reactivity to ASE may be useful to correct increased vulnerability to ASE observed in individuals with preexisting anxiety and depression-like stress behaviors. On the other hand, the CL induced decrease in adrenal stress reactivity of behaviorally normal animals suggests a potential risk of reducing the adaptive capacity of the organism under conditions of continuous light exposure.

## Introduction

The hypothalamic-pituitary-adrenal (HPA) axis is a key neuroendocrine system that underlies the body’s adequate response to environmental stressors. However, its dysfunction, accompanied by disturbances in the production of glucocorticoid hormones, contributes to the development of various stress-dependent diseases, including age-related, for example mental, metabolic, cognitive, cardiovascular, neurodegenerative, etc., the incidence of which is increasing (1–6). Increasing incidence of age-related diseases is largely due to rise of the proportion of aging individuals and life expectancy and also due to expansion of the range of stressful influences in the modern world (urbanization, environmental problems, local military conflicts, increased mental stress at work, etc.). Among the environmental factors effecting increase age-related diseases, great importance is given to disturbances of the light-dark schedule, particularly with increased illumination at night (7–14). Technological advances have transformed modern societies increasing the percentage of time that individuals spend indoors, where the illumination is much lower compared to bright sunlight or even cloudy daylight. At the same time, the illumination on the streets at night has sharply increased due to high levels of artificial light (11, 12, 15). Light pollution, irregular work and activity schedules, among other factors, can produce both transient and chronic disruption various physiological processes such as circadian rhythms of endocrine secretion and behavior, cognition, sleep-wake rhythm, alertness and performance, cardiovascular system, sympathetic activity, metabolic dysregulation, etc., and contribute to the development of age-related stress-dependent pathology or exacerbate it (7, 9, 10, 12, 15–19).

An extensive scientific material has been accumulated, according to which the basis of the negative impact of the light-dark schedule disturbances on physiological processes and health are damages in the circadian system. Thus, numerous literature data indicate the inhibitory effects of night lighting on pineal secretion of melatonin (MEL) (12, 13, 17, 20–23). A lot of attention has been paid to the functioning of the circadian system for the HPA axis in the normal conditions and under constant light (CL) but little is known regarding stress reactivity of the HPA axis in CL conditions.

So, it is assumed that the circadian regulation of glucocorticoid secretion is one of the best examples of bi-directional communication between the suprachiasmatic nucleus (SCN) of the hypothalamus, i.e. a central light-sensitive “master clock” and peripheral oscillators. SCN-mediated direct and indirect (through the subparaventricular nucleus and the dorsomedial hypothalamus) activation of corticotrophin-releasing hormone (CRH) and arginine-vasopressin (AVP) secretion from the hypothalamic paraventricular nucleus (PVN) controls the rhythmic release of adrenocorticotropic hormone (ACTH) from the pituitary gland, which in turn regulates the rhythmic production of glucocorticoids in the adrenal cortex (24–28). This pathway appears to be the main way that switches the light signal to the HPA axis, providing the basis for the circadian release of glucocorticoids with their zenith concentrations early in the morning and their nadir concentrations at night for humans and primates (25, 29, 30). In addition to this pathway, another indirect, extrahypophyseal autonomic pathway has also been identified. With this pathway the SCN transmits photic information to the adrenal medulla and from there through catecholamines to the adrenal cortex (25, 31, 32). Through this autonomic pathway, the SCN appears to predominantly modulate the sensitivity of the adrenal cortex to ACTH in a time-of-day manner (30, 33, 34), in particular by regulating the activity of the peripheral circadian clocks localized in the adrenal cortex (25, 32, 35).

In addition to controlling the circadian secretion of glucocorticoids with the help of the HPA axis and the extrahypophyseal nerve pathway, a number of authors assign certain importance in its regulation to the pineal hormone MEL (36, 37).

The publications on the functioning of the circadian system for the HPA axis under disruption of the light-dark schedule were carried out mainly on rodents that are nocturnal and differ significantly from humans and primates both in the functioning of the HPA axis and in the sensitivity of the optical system to light. This is probably why the data on the effect of CL on the function of the HPA axis are rather contradictory. Most studies point to disruption of the circadian rhythm of glucocorticoid secretion under CL conditions, accompanied mainly by an increase in their blood concentration in rats, mice, and hamsters (19, 38–45) At the same time, in single studies, along with glucocorticoids, an increase in the concentration of ACTH (40) or the absence of alterations in ACTH levels (32, 46) was noted. In contrast to previous works, a small number of studies have found no change (47, 48) or decrease in circulating glucocorticoids in response to CL in rodents (43, 49, 50). In most clinical studies, either no changes were noted in the levels of cortisol (CORT) in response to nighttime light exposure (51–55) or there has been its decrease (56, 57). The increase was observed only for a short time when applying pulses of bright light in the morning hours (52, 53) or as part of complex changes depending on the duration of light exposure (12). Effects of CL on HPA axis function largely appears to depend on the timing, the intensity, and possibly the duration of the light stimulus, as well as the spectral characteristics of light.

Unfortunately, we did not find any data on the features of HPA axis response to stress exposure under CL in the available literature, with the exception of the study that investigated the effect of an artificial light at night on the function of the adrenal cortex and the cardiovascular system in security guards working on the night shift (58). This study revealed an increase in the concentration of CORT and physiological prevalence of the vagal tone on the cardiocirculatory activity before and after the work shifts. We have previously presented data on the inhibitory effect of CL on the response of the HPA axis to the administration of AVP, which, to a certain extent, mimic the stress effect in monkeys (59). Unfortunately, all these investigations were performed on young individuals and did not affect the aging process, as well as individual differences. At the same time, the problem elucidation of stress reactivity of the HPA axis under disruption of the light-dark schedule remains relevant, especially during aging and depending on the features of stress behavior. Its resolve could be a source of information useful for the development of new approaches to prevent the increased vulnerability to stress and stress-dependent pathology noted in individuals with anxiety and depression-like stress behaviors (3, 6, 60–63). On the other hand, its solution is important to understand how chronic constant lighting affects the ability to adapt to stressful environmental factors.

The purpose of this study was to investigate the age-related and individual features of the HPA axis response to acute stress exposure in CL conditions, as well as the mechanisms underlying them, on the model of rhesus monkey females with control standard behavior and with depression-like and anxiety-like behavior using a two-hour restriction of their mobility (restraint, non-rigid immobilization) in metabolic cages as an acute psycho-emotional stress exposure (ASE), functional tests with CRH and AVP, and the study of circadian rhythms of plasma CORT and MEL in the basal period and on the background of CL. The planning of functional tests with the administration of CRH and AVP was carried out in order to assess the role of each of these neuropeptides in the mechanism of the identified age-related disorders in the HPA axis response to ASE in the conditions of chronic CL. As is well known and noted above (see page 2), CRH and AVP are secreted by neuroendocrine neurons of the hypothalamic PVN into the pituitary portal system. These neurons receive a large number of diverse neural signals from various parts of the brain, which contribute to the endocrine response to stress exposure. CRH and AVP interact with specific receptors on corticotrophs of the anterior pituitary gland (CRH with CRHR1 and AVP with AVP1b, respectively) to induce ACTH secretion into the general circulation. ACTH, after binding to type 2 melanocortin receptor in the adrenal cortex, activates a signaling cascade that usually leads to *de novo* biosynthesis and release of glucocorticoids (predominantly CORT in humans and nonhuman primates and corticosterone in most rodents) (6, 30). In addition to stress, as noted above (see page 2) CRH and AVP play an important role in the rhythmic release of ACTH and CORT receiving information from the SCN about the environment illumination (28, 30). The administration of CRH or AVP, as in the case of ASE, is also accompanied by an increase in the secretion of ACTH and CORT in young and old monkeys (6). Moreover, significant differences in ACTH response to CRH and AVP tests were found in nonhuman primates differing in stress behavior, with a higher rise in ACTH secretion in individuals with anxiety and depression-like behavior, similar to intergroup differences in ACTH response to ASE in old female rhesus monkeys, and in contrast to the absence of between-group differences in ACTH response to ASE in young monkeys (6, 61).

We demonstrated for the first time an inhibitory effect of CL on the response of the adrenal cortex to acute stress exposure in all individuals, regardless of age and behavioral patterns, the mechanisms of which were age-dependent: due to inhibition of pituitary secretion of ACTH in young animals and mainly without affecting it, in old individuals.

## Material and methods

### Animals

Thirty five young adult (5–8 years) and 23 old (21–33 years) healthy female rhesus monkeys (*Macaca mulatta*) were used in the experiments. The monkeys originated from the Adler monkey colony (Research Institute of Medical Primatology, Sochi-Adler, Russia). The animals usually were housed in open enclosures (housing 10-15 or 40-50 individuals of various ages, including newborns and elderly animals) or cages designed for group housing (3-5 individuals). During the experiment, the animals were moved into individual metabolic cages in a separate room with narrow windows, natural illumination (usually from 06.00 h to 18.00 h) and controlled temperature (25-28°C). Experimental and control animals were kept in different rooms. The experiments were carried out in summer time (June-August) when ovarian cycles are not typical for this species of laboratory primates. The animals were fed pellets prepared according to the technique of Altromin (Lage). The pellet diet was complemented with fresh vegetables, fruits and water *ad libitum*. Prior to the experiments, the animals were adapted to the conditions of separate housing and to the procedure of blood sampling for 4 weeks. During the adaptation period, the lighting in the rooms with experimental and control animals usually was about 60-130 lux. However, artificial lighting was additionally turned on during the period of room cleaning and experimental procedures on cloudy days for a short period of time (see Methods section below).

All procedures were obtained approval of the Ethical Committee of the Research Institute of Medical Primatology (Sochi), and all operations in this study were conducted in accordance with the guidelines of the European Convention for the Protection of the Vertebrate Animals Used for Experimental and Other Scientific Purposes” (Strasbourg, 18.III.1986), Directive 2010/63/EU of the European Parliament and the Council of 22 September 2010 (on the protection of animals used for scientific purposes).

A more detailed description of the conditions for keeping the monkeys in the nursery, the assessment of their health and behavior, as well as the preparing animals for experiments were carried out according to standard methods, as described earlier (64).

#### The experimental groups of monkeys

The data presented in the article were obtained as a result of physiological experiments (acute psycho-emotional stress modeling, experiment with administration of CRH, and experiment with administration of AVP) for 3 years - from 2019 to 2021. In addition, in all animals, the features of the effect of CL on the circadian rhythms of CORT and MEL were studied every year. In the experiments of each year, experimental and control groups of animals were distinguished, as well as the basal period and the actual experimental period, i.e. period of CL and experimental period for control animals with normal light-dark schedule (standard light, SL).

The animals’ behavior was recorded while they were housed in the metabolic cages, both during the period of adaptation, and throughout experimentation. Depending on behavioral features, both young and old animals were divided into two groups: with healthy active adaptive behavior (standard behavior, SB) and with maladaptive depression-like and anxiety-like behavior (DAB). In 2019, 11 animals with DAB and 6 animals with SB were used in experiments; in 2020 - 12 animals with DAB and 10 animals with SB; in 2021 - 8 animals with DAB and 11 animals with SB.

Analysis of the life history of the experimental animals revealed that 2 animals with DAB (№ 40176 - young, 34347- old) and 1 control old animal (№35721) were exposed to severe stress in early childhood (maternal deprivation due to maternal death in the period from 1 month to 9 months) and growing up separately from adult individuals, in the so-called “nursery” in individual cages until age 1.2 years, and then were kept in the cage designed for group housing, together with other immature animals, deprived of mother, and previously lived in the “nursery.”

### Methods

#### Experiment with acute stress exposure (ASE)

Twenty two female rhesus monkeys were used in the experiments. The group of young experimental animals included 8 individuals (7.5 ± 0.5 years, 6.0 ± 0.5 years); the group of old experimental animals also included 8 individuals (26.3 ± 1.6 years, 6.1 ± 0.5 kg). The group of control animals included 6 young monkeys (8.0 ± 0.45 years, 6.0 ± 0.2 kg). In this experiment, 8 experimental animals with DAB (young – 5, old – 3), 8 experimental animals with SB (young – 3, old – 5), 4 control animals with DAB, and 2 control animals with SB were used.

##### Basal period

After adaptation period, all animals were subjected to ASE: moderate restraint (non-rigid immobilization, restriction of mobility) in a metabolic cage for two hours, as described earlier (61). Restraint was achieved by using a conventional squeeze board to press the animal to the front wall of the metabolic cage. The body and extremities of the animal were not tightly immobilized. Animals were subjected to the stressor at 15.00 h. Blood samples were taken before restraint (0) and 15, 30, 60, and 120 min during application of the stressor, and at 240 min, i.e., 2 h after termination of the stressor exposure.

##### Constant light (CL) period and experimental period for control animals (standard light, SL)

The adaptation and basal periods were followed by the actual experimental period, during which artificial light (330-400 lux) was turned on in the room with young and old animals of the experimental groups during the day and night continuously for 8 weeks (period of CL). At the same time in the room with young and old animals of the control groups, the light remained the same, i.e. standard (about 60-130 lux) with predominantly natural light during the day and darkness at night (period of SL). It should be noted that during the daytime, the experimental animals were additionally exposed to natural light, similar to that in the room with the control group of animals, that is, 60-130 lux. For chronic CL we used white light-emitting diode lamps with a wavelength of 400-838 nm and with a predominant wavelength range of 513-700 nm (green, yellow, orange, red colors) intended for residential, office, and commercial premises (LED lamp “Navigator” 71 302 NLL-G-T8-18-230-4K-G13, Limited Liability Company “TM Navigator”, Moscow, Russia; made in China – Xiamen Neex Optical Electronic Technology CO., LTD). After 7 weeks of the experimental period all experimental and control animals were exposed ASE in the same way as in the basal period.

#### Experiments with CRH administration

Nineteen female rhesus monkeys were used in the experiments, including 6 young experimental (8.0 ± 0.45 years, 5.96 ± 0.24 kg), 5 young control (6.0 ± 0.37 years, 5.0 ± 0.4 kg), 5 old experimental (27.6 ± 0.4 kg) 2.5 years, 5.0 ± 0.3 kg) and 3 old controls (18.0 ± 0 years, 6.7 ± 0.8 kg). In this experiment, 5 experimental animals with DAB (young – 4, old – 1), 6 experimental animals with SB (young – 2, old – 4), 5 control young animals (1 - with DAB and 4 with SB) and 3 control old animals (1 - with DAB and 2 with SB) were used. All experimental and control animals twice in basal period and after 8 weeks, respectively CL period and SL period were injected with CRH in 0.9% NaCl (Сorticotropin Releasing Factor of human, rat, Sigma; intravenously in a dose of 1 μg/kg body weight). Blood samples were taken before (0 min) and 15, 30, 60, 120, and 240 min after the drug administration. Animals were subjected to the injection of CRH at 15.00 h.

#### Experiments with AVP administration

Seventeen female rhesus monkeys were used in the experiments, including 5 young experimental (7.6 ± 0.6 years, 6.2 ± 0.6 kg), 5 young control (6.6 ± 0.4 years, 5.7 ± 0.4 kg), 4 old experimental (24.2 ± 1.7 years, 6.7 ± 0.8 kg) and 3 old controls (29.3 ± 2.7 years, 5.7 ± 0.7 kg). In the experiment, 11 animals with DAB and 6 animals with SB were used. All experimental and control animals twice in basal period and after 6 weeks, respectively CL period and SL period, were injected with AVP in 0.9% NaCl (Arg8-Vasopressin, MP Biomedicals, LLC, France) intravenously in a dose of 1 μg/kg body weight. Blood samples were taken before (0 min) and 15, 30, 60, 120, and 240 min after the drug administration. Animals were subjected to the injection of AVP at 15.00 h.

#### Experiments to study the circadian rhythms of CORT and MEL

To assess the circadian rhythms of CORT and MEL, blood samples were taken from 12 young and 10 old experimental animals, 10 young and 6 old control animals in the basal period and in the CL or SL period (5 weeks) at 09.00 h, 15.00 h, and 22.00 h.

#### Hormone measurements

All blood samples were taken from cubital vein of the animals. Blood samples were collected in chilled tubes with EDTA (10.0 mg per 1 ml of blood) as the anticoagulant. At each time point 1.0-2.0 ml of blood was taken. Blood samples were immediately centrifuged at 2000g at +4°C, plasma stored at −70°C for later analysis. Plasma levels of CORT, ACTH, and MEL were measured by immunoenzyme assay using standard hormone kits (AlkorBio, Russia for total CORT; Biomerica Inc., USA for ACTH; IBL international GmbH, Germany for MEL). The ELISA method for the determination of MEL included a stage of preliminary purification on columns with a sorbent. The sensitivity of the assay for CORT was 10.0 nmol/l. The intra-assay and inter-assay variation coefficients (C.V.) for CORT did not exceed 10 and 15%, respectively. The sensitivity of the assay for ACTH was 0.22 pg/ml. The intra-assay and inter-assay variation coefficients for ACTH did not exceed 8 and 10%, respectively. The sensitivity of the assay for MEL was 1.6 pg/ml. The intra-assay and inter-assay variation coefficients for MEL did not exceed 12 and 17%, respectively.

#### Statistical analysis

The experimental values are presented in tables and figures as means ± S.E.M. The statistical comparisons of hormone level differences at various time intervals after the start of exposure in comparison with the initial levels of the same hormones and between the corresponding values of hormones under CL and SL and in the basal period and also the age and behavioral group differences were performed using one- and two-way analysis of variances (ANOVA) including *post hoc* Tukey’s honest significant difference test for paired comparisons (Statistics 10 software package, Stat Soft. Inc., USA). The amplitude of the circadian rhythm of CORT was calculated as the difference between the hormone concentrations at 09.00 and 22.00 h, and the amplitude of the circadian rhythm of MEL was calculated as the difference between its concentrations at 22.00 (or 21.00) and 09.00 h. The areas under curves representing hormone concentration as a function of time (0-240 min, response area) were calculated using the trapezium formula.

## Results

### Effect of chronic CL on the response of the HPA axis to ASE and its possible mechanisms in young female rhesus monkeys

#### Effect of CL on the HPA axis stress responsiveness

**Figure 1:**
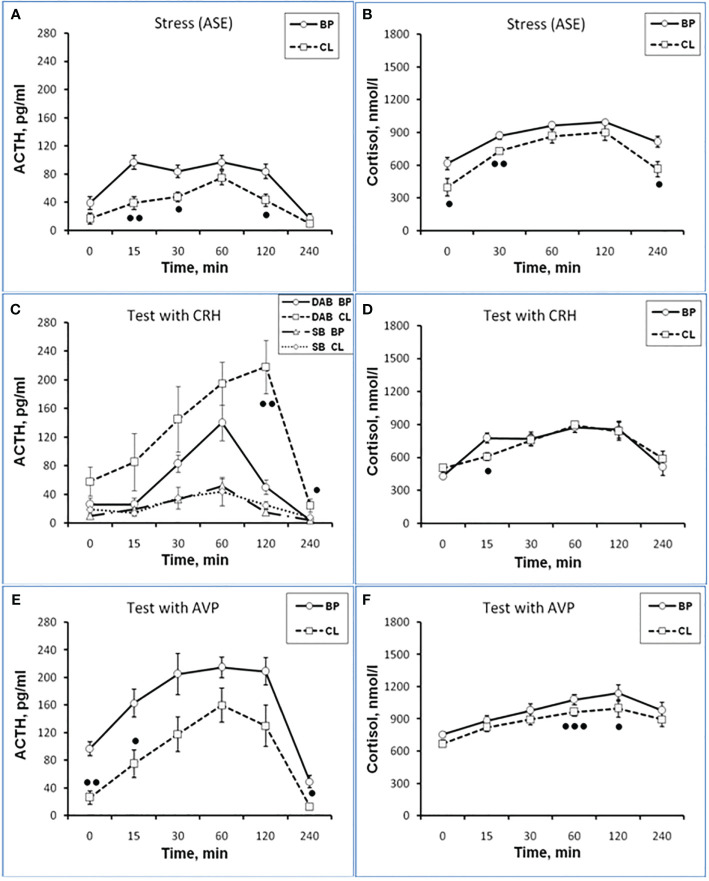
Dynamics of ACTH and CORT levels in peripheral blood plasma in young experimental female rhesus monkeys in the basal period (BP) and on the background of chronic constant lighting (CL) in response to ASE **(A, B)**, administration of CRH (**C**: -Δ- the animals with SB and -o- with DAB in the basal period; …o… animals with SB and □ with DAB at CL period) **(D)**, and AVP **(E, F)** (mean ± S.E.M.). ●p <0.05; ●●p<0.01; ●●●p<0.001 - vs. relative values in the basal period.

**Figure 2:**
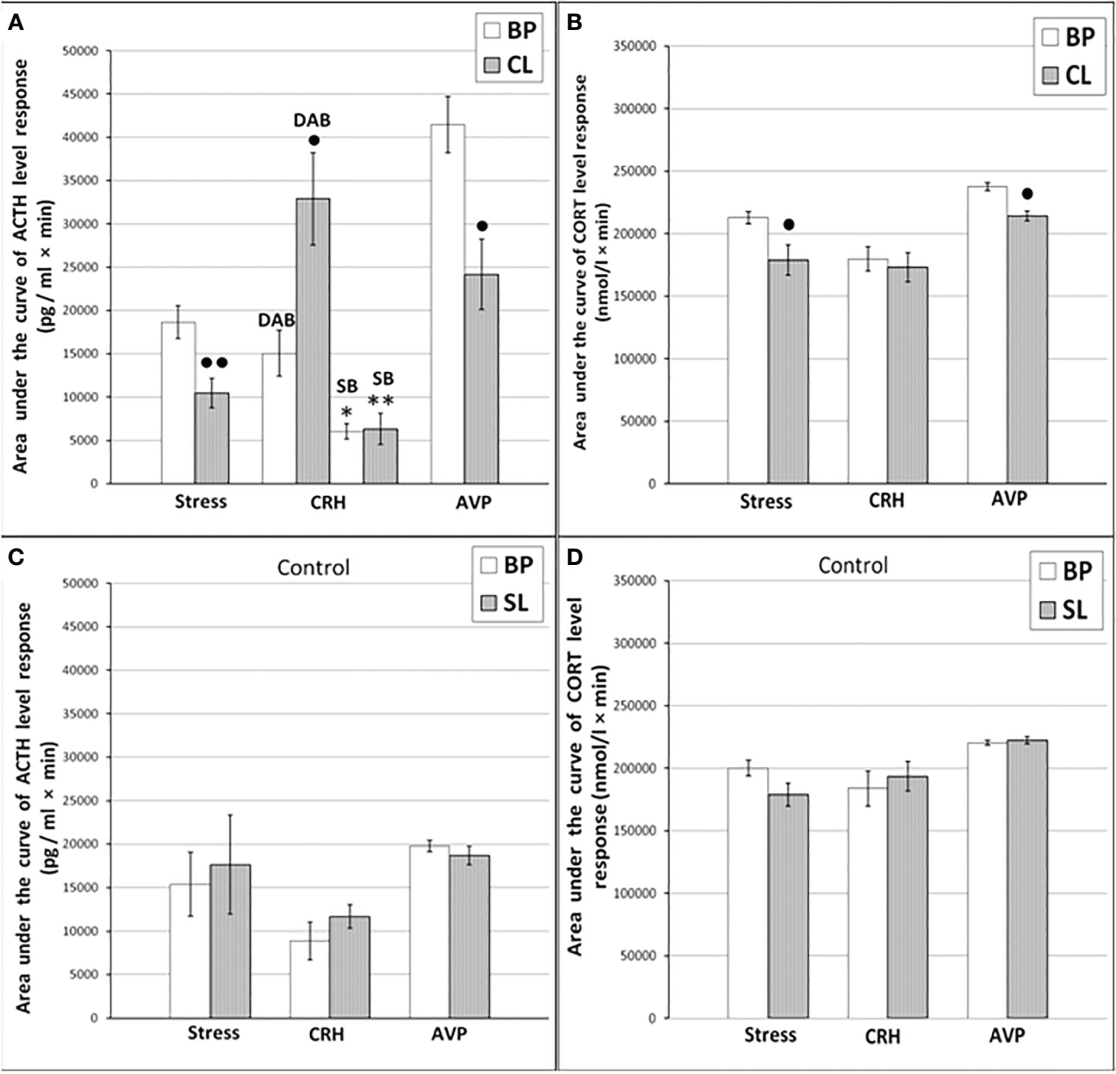
The areas under the concentration curves of ACTH and CORT in response to ASE, administration of CRH (including animals with SB and DAB for ACTH) and AVP in young experimental **(A)** ACTH, **(B)** CORT and control **(C)** ACTH, **(D)** CORT female rhesus monkeys in basal period and on the background of CL or standard lighting (SL) (mean ± S.E.M.). ●p <0.05; ●●p<0.01 - vs. the values in basal period; * p <0.05; ** p<0.01 - vs. the relative values in the animals with DAB.

**Figure 3:**
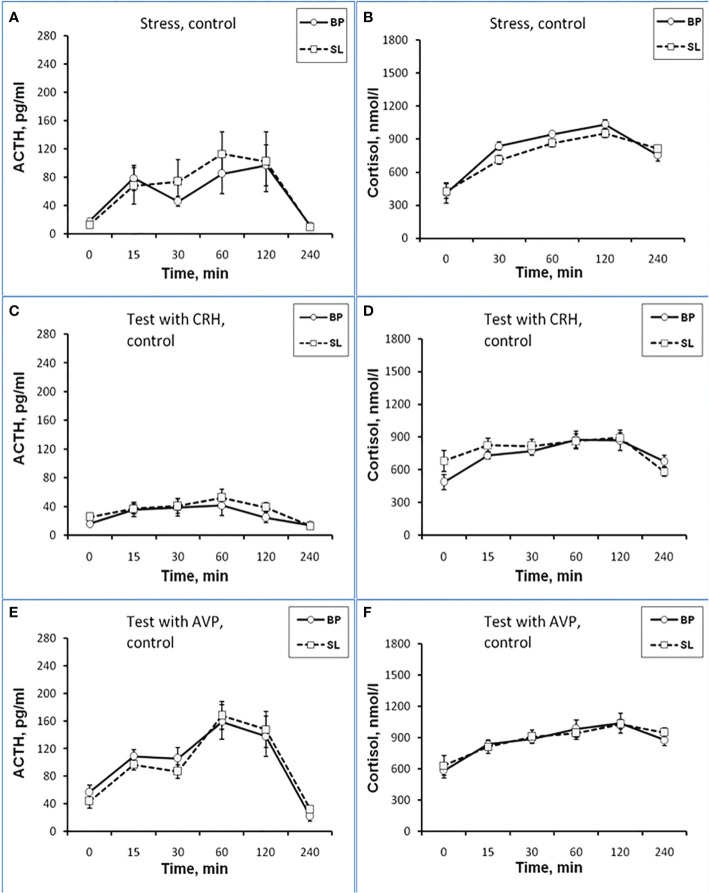
Dynamics of ACTH and CORT levels in peripheral blood plasma in young control female rhesus monkeys in the basal period (BP) and on the background of standard lighting (SL) in response to ASE **(A, B)**, administration of CRH **(C, D)**, and AVP **(E, F)** (mean ± S.E.M.).

Dynamics of ACTH and CORT levels in young female rhesus monkeys (without division by type of behavior) in response to acute stress exposure (ASE) under basal conditions and after 7 weeks of exposure to CL indicates the inhibitory effect of CL on the magnitude of the rise in ACTH and CORT concentrations (**Figures 1A, B**). In addition, CL reduced the initial levels of СORT (**Figure 1B**). The areas under curves representing dynamics of ACTH and CORT in response to ASE on the background of CL were also significantly lower than in basal period (**Figures 2A, B**). At the same time, there were no significant changes in both the areas of response of ACTH and CORT concentrations (see **Figures 2C, D**) and the dynamics of their response (**Figures 3A, B**) to ASE in animals of the control group in the basal period and on the background of standard lighting. A decrease in the magnitude of the rise in ACTH in response to ASE under conditions of CL was statistically significant for both animals with DAB and SB (**Table 1**). The decrease in the magnitude of the rise in the level of СORT on the background of CL was characteristic of animals with DAB (**Table 1**) and was also observed in animals with SB. Thus, the response area of CORT in three experimental animals with SB in the basal period was 227940, 204960 and 191700 nmol/l×min but under CL it was 214200, 130350 and 124200 nmol/l×min, respectively.

**Table 1:** Dynamics of ACTH and CORT concentration in peripheral blood plasma and the area under the curve of ACTH and CORT response (0-240 min) to ASE in young experimental female rhesus monkeys with DAB and SB in the basal period and under CL (mean ± S.E.M.).

Experiment conditions	Time, min						S 240
	0	15	30	60	120	240	
DAB (n=5)							
ACTH, pg/ml							pg/ml×min
Basal period	52 ± 10	102 ± 20 #	137 ± 15 ##	141 ± 18 #	111 ± 10	23 ± 10	25320± 2160
CL	23 ± 10	45 ± 10 ●	53 ± 7 ●●	97 ± 10 #	54 ± 10 ##●	12 ± 3	12775± 1900●
CORT, nmol/l							nmol/l×min
Basal period	715 ± 66	–	870 ± 40 ##	967 ± 7 #	1007 ± 30 ##	880 ± 33	218617± 4959
CL	435 ± 88 ●	–	764 ± 18 #●	924 ± 36 #	992 ± 50 #	620 ± 98 ●	192450 ± 26100● (S.E.M.)
SB (n=3)							
ACTH, pg/ml							pg/ml×min
Basal period	18 ± 3	88 ± 20	111 ± 36	53 ± 10	38 ± 9	5 ± 1#	14480± 2300
CL	5 ± 3 ●	28 ± 10	39 ± 10	39 ± 10	25 ± 9	5 ± 1	6552± 1590●

# p < 0.05; ## р < 0.01 – vs. the corresponding values before ASE (0 min); ● p<0.05; ●● р< 0.01 – vs. the values in basal period.

Thus, the obtained experimental data indicate the inhibitory effect of CL on the response of the HPA axis to ASE by suppressing both ACTH and CORT secretion in all experimental young animals, regardless of behavioral characteristics. In order to study the possible mechanisms of the inhibitory effect of CL on the HPA stress reactivity, we performed functional tests with the administration of CRH and AVP, the main central drivers of the HPA axis, and also evaluated the effect of CL on the circadian rhythm of the activity of the adrenal cortex and the pineal gland.

#### Reaction the HPA axis to administration of CRH

It was found that in the group of experimental animals (without subdividing them according to the type of behavior), the concentration of ACTH in response to CRH administration on the background of CL did not statistically significantly differ from the corresponding values in the same animals under basal period. However, in the group there was a large individual variability in the response of ACTH to CRH, both on the background of CL and under basal period (**Table 2**). At the same time, a certain pattern was revealed, according to which, according to the magnitude of the rise in the level of ACTH to the administration of CRH, animals of the experimental group could be divided into 2 subgroups: (1st) with a high response (3 individuals with DAB) and (2nd) with a low response (3 individuals with SB) (**Figure 1C**). In animals with DAB, the concentration of ACTH on the background of CL 120 and 240 min after the administration of CRH was statistically significantly higher compared to similar values in the basal period. At the same time, the magnitude of the increase in the level of ACTH in animals with SB on the background of CL remained the same as in the basal period (**Figure 1C**). The response area of ACTH concentration in animals of the 2nd subgroup was significantly lower compared with the corresponding values in animals of the 1st subgroup both in the basal period and on the background of CL (**Figure 2A**). In control animals, there were no statistically significant changes in the dynamics of ACTH concentration and in response areas to the test with CRH on the background of SL **(** **Figures 2C** and **3C**).

**Table 2:** Dynamics of ACTH concentration (pg/ml) in peripheral blood plasma and the area under the curve of ACTH response (0-240 min) to CRH administration in young experimental female rhesus monkeys in the basal period and under CL (mean ± S.E.M.).

Experiment conditions	Time, min						S 240, pg/ml×min
	0	15	30	60	120	240	
Basal period	18 ± 6	23 ± 5	62 ± 10 ##	92 ± 20 #	34 ± 9	4 ± 0,5	10624± 2340
CL	39 ± 10	50 ± 20	90 ± 30	120 ± 38 #	120 ± 46	16 ± 6	19610± 6450

# p < 0.05; ## р < 0.01 – vs. the corresponding values before CRH administration (0 min).

An analysis of the dynamics of the CORT level in female rhesus monkeys in response to the administration of CRH under basal conditions and on the background of CL revealed no significant differences in general, except for the CORT concentration 15 min after the administration of CRH. It was statistically significantly lower on the background of CL than in the basal period (**Figure 1D**). There were no statistically significant differences in the areas of the response of CORT on the background of CL and in the basal period (**Figure 2B**). In addition, there were no essential differences in the dynamics of CORT concentration and the CORT response area on the background of CL and in the basal conditions in control animals (**Figures 2D** and **3D**).

#### Reaction the HPA axis to administration of AVP

The dynamics of ACTH level in experimental and control young female rhesus monkeys in response to the administration of AVP in the basal conditions and on the background of CL and SL is shown in **Figures 1E** and **3E**. As can be seen, the CL led to a pronounced decrease in the magnitude of the rise in ACTH concentration in young primates before the start of AVP administration (0 min), as well as 15 and 240 min after its administration (**Figure 1E**). In addition, in response to the administration of AVP, a statistically significant decrease was observed in the ACTH response area (**Figure 2A**). At the same time, in control animals, in response to AVP, there were no statistically significant differences in the ACTH response in the basal period and on the background of SL (**Figures 2C** and **3E**).

The magnitude of the concentration of CORT rise in the test with AVP in the animals on the background of CL 60 and 120 min after administration of the drug as well as the area of the CORT response, were significantly lower than in the basal period (**Figures 1F** and **2B**). At the same time, the dynamics of CORT concentration and the response area of CORT in control animals in response to the injection of AVP practically did not differ from similar values in the basal period (**Figures 2D** and **3F**).

Thus, the reaction of the HPA axis to the test with AVP on the background of CL was significantly lower compared to the basal period and was similar to the response of the HPA axis to ASE. It should be noted that in this experiment the majority of experimental (4 individuals out of 5) and control (3 individuals out 5) animals belonged to the DAB type. Therefore, all of the above in the experiment with AVP primarily applies to animals with DAB.

#### Blood plasma CORT and MEL at different times of the day

**Figure 4:**
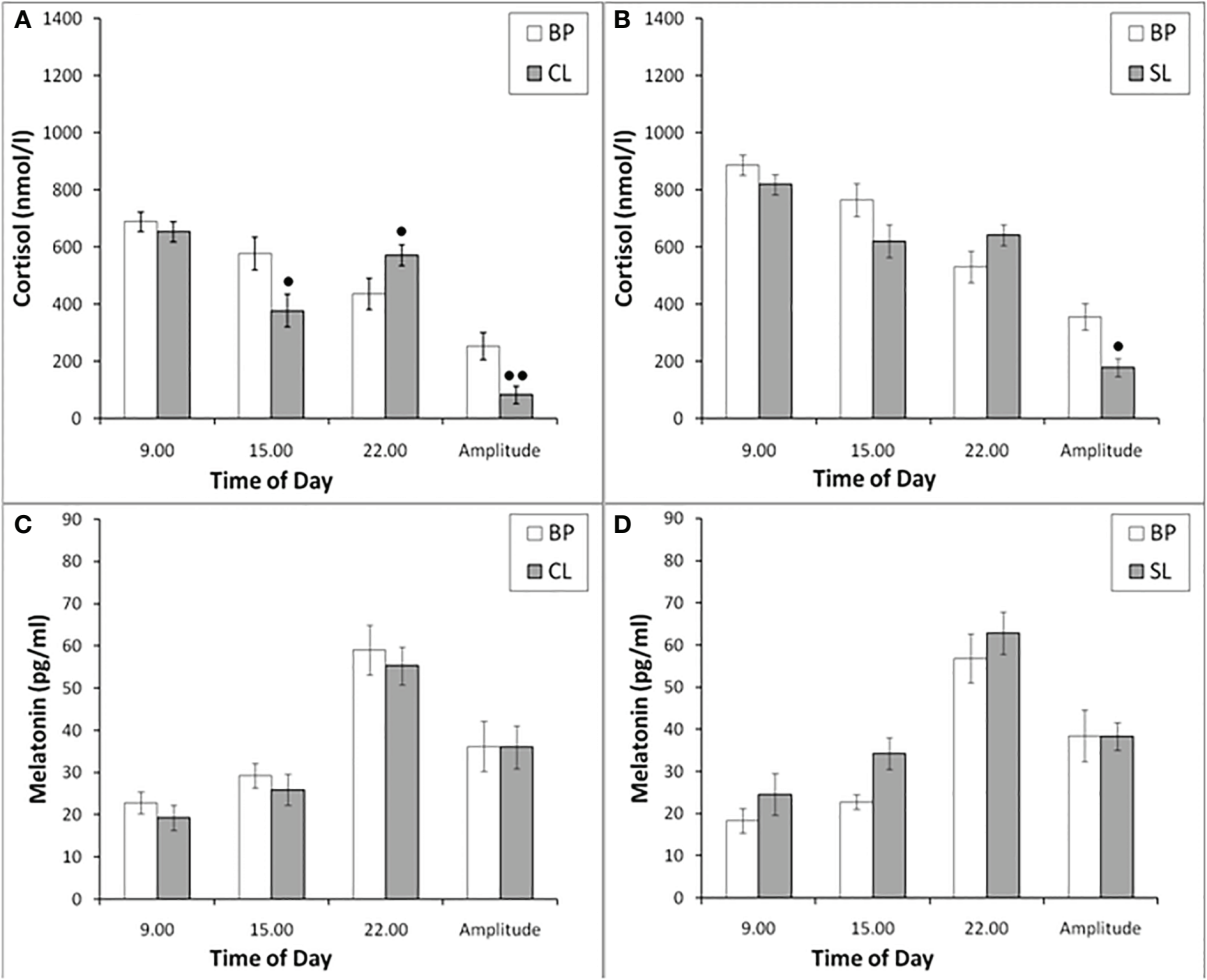
The concentration of CORT and MEL at different times of the day and the amplitude of their circadian rhythms in behaviorally combined groups of young experimental (n=12; **A, C**) and control (n=10; **B, D**) female rhesus monkeys in the basal conditions and under the CL or SL conditions (mean ± S.E.M.). ● p<0.05; ●● р< 0.01 vs. the values in basal period.

The blood plasma CORT at different times of the day in experimental female rhesus monkeys (combined group of animals with SB and DAB) showed a pronounced decrease at 15.00 compared to similar values in the basal period (**Figure 4A**). In addition, in experimental animals, an increase in the level of CORT at 22.00 was noted with a corresponding decrease in the amplitude of the circadian rhythm (**Figure 4A**). In control animals, there was a tendency to a decrease in the level of CORT at 15.00 and an increase at 22.00 on the background of SL, which, however, were accompanied by a statistically significant decrease in the amplitude of the circadian rhythm (**Figure 4B**). Apparently, CL is not a specific factor that induces an increase in the concentration of CORT at night, but its presence enhances this process.

The plasma MEL at different times of the day in experimental and control female rhesus monkeys (combined groups of animals with SB and DAB) demonstrated no significant changes at 09.00, 15.00 and 22.00, as well as in the amplitude of its circadian rhythm on the background of CL, as well as on the background of SL (**Figures 4С, D**).

### Effect of chronic CL on the response of the HPA axis to ASE and its possible mechanisms in old female rhesus monkeys

#### Effect of CL on the HPA axis stress responsiveness

**Figure 5:**
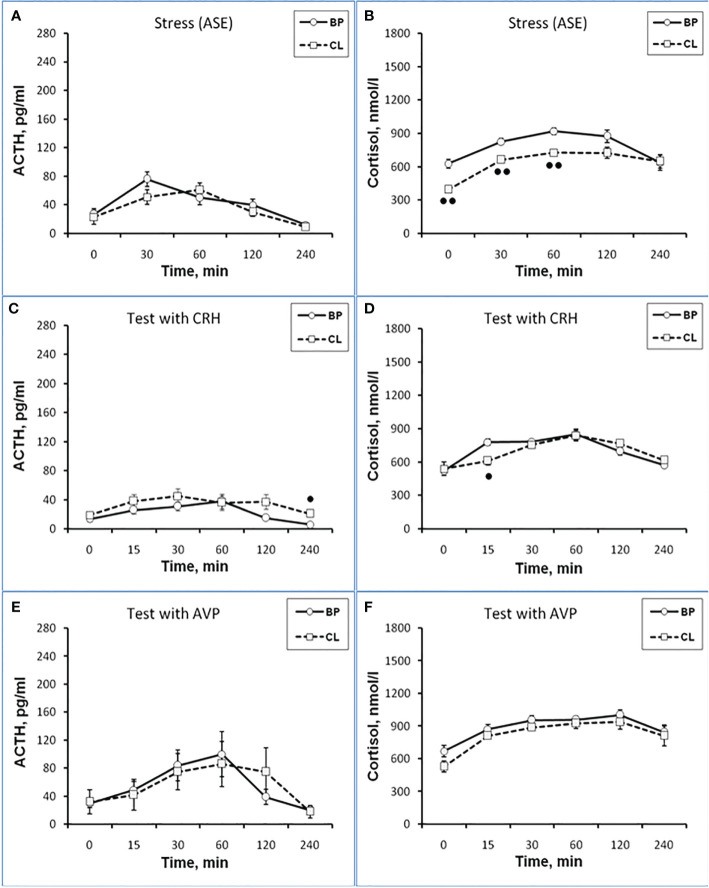
Dynamics of ACTH and CORT levels in peripheral blood plasma in old experimental female rhesus monkeys in the basal period (BP) and on the background of chronic constant lighting (CL) in response to ASE **(A, B)**, administration of CRH **(C, D)**, and AVP **(E, F)** (mean ± S.E.M.). ●p <0.05; ●●p<0.01 - vs. relative values in the basal period.

**Figure 6:**
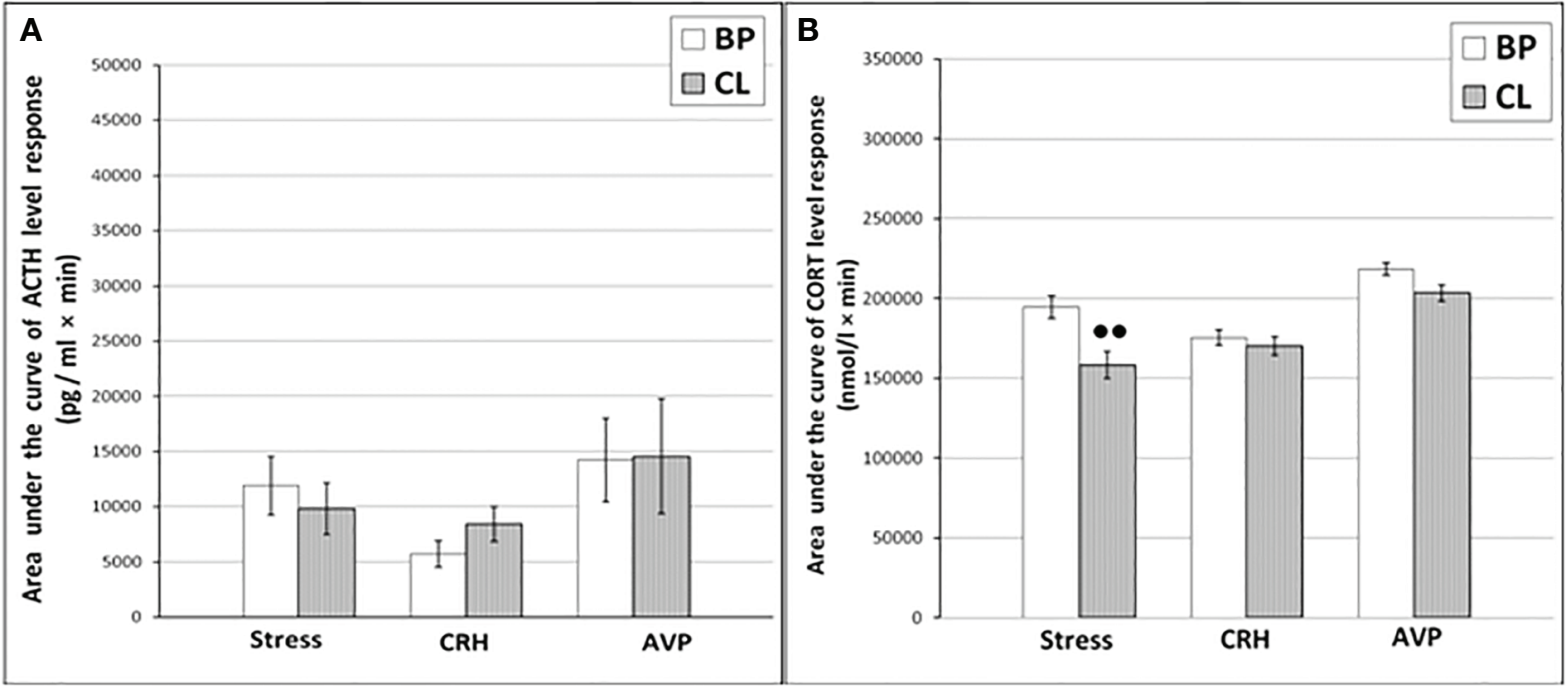
The areas under the concentration curves of ACTH **(A)** and CORT **(B)** in response to ASE, administration of CRH and AVP in old experimental female rhesus monkeys in basal period and on the background of CL (mean ± S.E.M.) ●●p<0.01 - vs. the values in basal period.

The CL had no significant effect on the dynamics of ACTH concentration and the response area of ACTH to ASE in the behaviorally combined group of old animals (**Figures 5A** and **6A**) and also in the animals with SB (**Table 3**). However, a slight decrease in the concentration of ACTH was observed in two animals with DAB (**Table 3**). At the same time, in contrast to ACTH, the magnitude of the rise in CORT level in response to ASE on the background of CL, including the response area of CORT, was significantly lower than in the basal period (**Figures 5B** and **6B**).

**Table 3:** Dynamics of ACTH concentration (pg/ml) in peripheral blood plasma and the area under the curve of ACTH response (0-240 min) to ASE in old female rhesus monkeys with SB and DAB in the basal period and under CL (mean ± S.E.M.).

Experiment conditions	Time after the onset of ASE, min						S240, pg/ml×min
	0	15	30	60	120	240	
SB (n=5)							
Basal period	19 ± 6	30 ± 8	50 ± 6	31 ± 8#	29 ± 6	9 ± 1	7960± 680
CL	9 ± 3	26 ± 10	46 ± 10	44 ± 10#	25 ± 4#	9 ± 1	7190± 2100
DAB (n=2)							
BP-30858	66	150	140	90	74	27	24024
BP-31868	25	145	80	105	60	12	19608
CL-30858	80	130	66	105	330	80	18288
CL-31868	33	70	60	105	56	12	15048

#p < 0.05 – vs. the corresponding values before ASE (0 min).

Thus, in old animals, in contrast to young animals, there was no significant decrease in the ACTH response to ASE induced by CL. At the same time, CL induced a significant decrease in both the basal level of CORT and the magnitude of its rise in response to ASE for all animals, regardless of behavior. Apparently, CL in old animals inhibits the magnitude of the rise in the concentration of CORT in response to ASE, mainly without affecting the secretion of ACTH. In order to elucidate the mechanisms underlying the inhibitory effect of CL on the stress reactivity of the adrenal cortex in old female rhesus monkeys, as in the case of young animals, we studied the response of ACTH and CORT to tests with the administration of CRH and AVP, as well as the circadian rhythms of CORT and MEL in basal conditions and on the background of CL.

#### Reaction the HPA axis to administration of CRH

**Figure 7:**
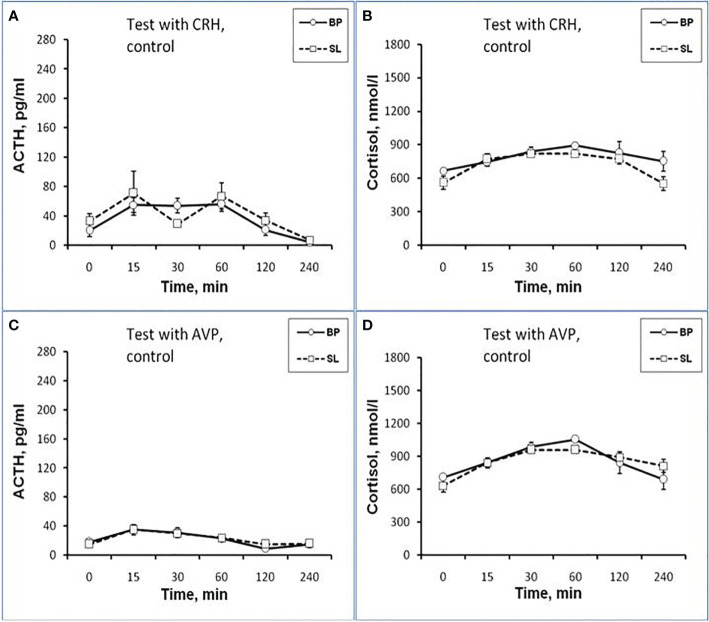
Dynamics of ACTH and CORT levels in peripheral blood plasma in old control female rhesus monkeys in the basal period (BP) and on the background of standard lighting (SL) in response to administration of CRH **(A, B)** and AVP **(C, D)** (mean ± S.E.M).

As shown by a functional test with CRH, in old experimental female rhesus monkeys on the background of CL, there were no significant changes in the magnitude of the rise in ACTH concentration compared to the basal period, except for the point of 240 min, where a statistically significant increase in the level of ACTH was noted (**Figure 5C**). There were no statistically significant changes in the ACTH response area (**Figure 6А**). In control animals, there were also no significant changes in the ACTH response to the test with CRH on the background of standard illumination (**Figures 7А** and **8A**). The response of CORT to the administration of CRH on the background of CL in old female rhesus monkeys, as in the case of young females, did not undergo significant changes, except for the significantly lower values of the CORT level 15 min after the administration of CRH, compared to the response of CORT in basal period (**Figure 5D**). The response area of CORT to the injection of CRH did not undergo statistically significant changes in experimental animals (**Figure 6B**). There were no significant changes in the dynamics of the plasma CORT and the response area to the administration of CRH in the control group of animals on the background of SL (**Figures 7В** and **8B**).

**Figure 8:**
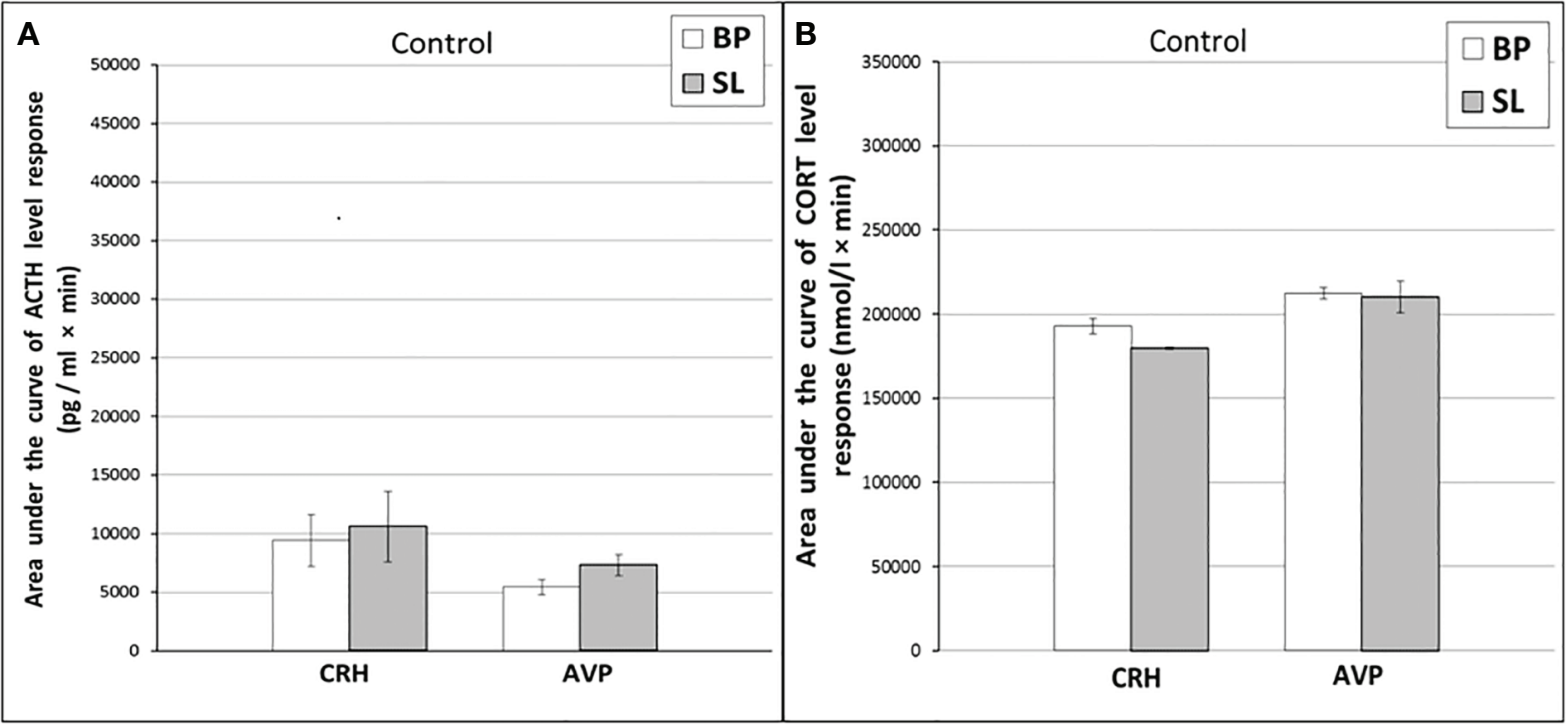
The areas under the concentration curves of ACTH and CORT in response to administration of CRH **(A, B)** and AVP **(A, B)** in old control female rhesus monkeys in basal period and on the background of standard lighting (SL) (mean ± S.E.M.).

#### Reaction the HPA axis to administration of AVP

In the experiment, 5 old female rhesus monkeys were used (3 individuals with DAB and 2 individuals with SB). In contrast to young animals, the CL did not lead to a significant decrease in the magnitude of the rise in ACTH concentration and the area response to the administration of AVP in old female rhesus monkeys (**Figures 5E** and **6A**). There were no significant changes in the ACTH response to the administration of AVP in control old animals (**Figures 7С** and **8A**). At the same time, it should be noted that polymorphism was observed in the reaction of ACTH to the administration of AVP under CL. So, in 3 old experimental animals, as well as in young female rhesus monkeys, a decrease in the magnitude of the increase in ACTH concentration was observed in response to the administration of AVP. Thus, the response areas of ACTH concentration in the basal period and under CL were respectively: 20880, 20784 and 5602 pg/ml×min and 4512, 10728 and 3912 pg/ml×min. Similar to the dynamics of the level of ACTH, the concentration of CORT in the peripheral blood plasma in old experimental female rhesus monkeys in response to the AVP administration under CL did not undergo significant changes compared to the basal period (**Figures 5F** and **6B**). There were no significant changes in the response of CORT to AVP in control animals on the background of SL (**Figures 7D** and **8B**). It should be noted that in old animals with a pronounced decrease in the magnitude of the rise in plasma ACTH on the background of CL, there was also a slight decrease in the level of CORT. Thus, the response area of ACTH for 240 min in the animals under discussion was 225600, 203520, 214800 nmol/l×min, respectively, in the basal period and 199440, 182160 and 189600 nmol/l×min under CL.

#### Blood plasma CORT and MEL at different times of the day

**Figure 9:**
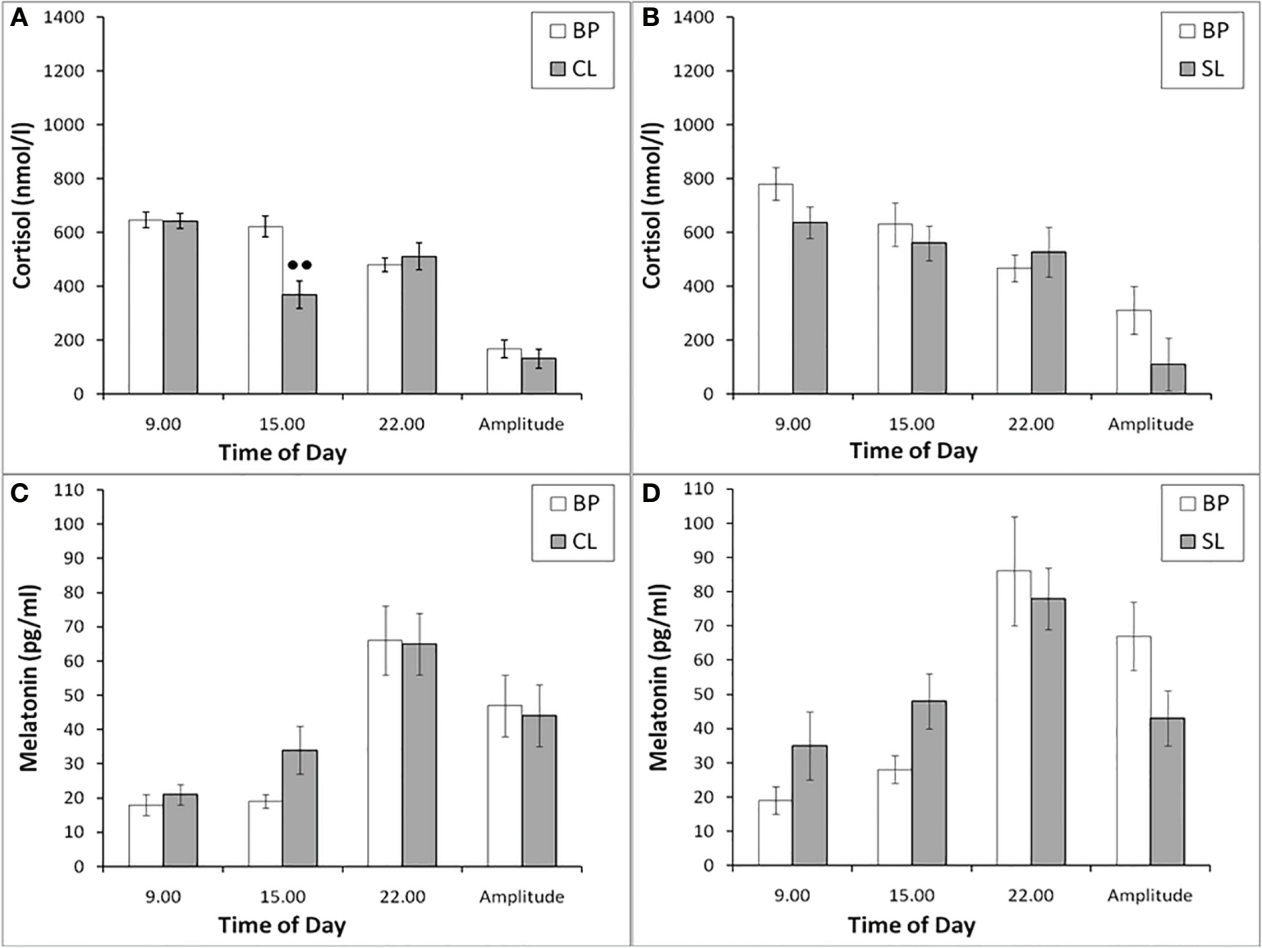
The plasma concentration of CORT and MEL at different times of the day and the amplitude of their circadian rhythms in behaviorally combined groups of old experimental (n=10; **A, C**) and control (n=6; **B, D**) female rhesus monkeys in the basal conditions and under the CL or SL conditions (mean ± S.E.M.). ●● р< 0.01 vs. the values in basal period.

As in young female rhesus monkeys, the concentration of CORT in old animals on the background of CL was statistically significantly lower compared to the basal period at 15.00 and was characterized by a tendency to increase at 22.00 (**Figure 9A**). In control animals, significant changes in the concentration of CORT at different times of the day on the background of SL were not detected (**Figure 9B**). Significant changes in the circadian rhythm of MEL on the background of CL in old female rhesus monkeys, like young females, were not revealed either in comparison with the basal period or in comparison with control animals (**Figures 9C, D**).

## Discussion

As a result of the research, we demonstrated for the first time an inhibitory effect of CL on the response of the adrenal cortex to ASE applied in the afternoon (start at 15.00) in young mature and old female rhesus monkeys. Inhibition of the function of the adrenal cortex under CL was characteristic of both for animals with DAB and with control SB. Although the inhibition of the function of the adrenal cortex was detected in all the examined animals, regardless of age and behavior, the mechanism of the inhibitory effect of CL on the stress reactivity of the adrenal cortex, apparently, is age-dependent. So, young mature female rhesus monkeys were characterized by an inhibitory effect of CL both on the magnitude of the rise in CORT secretion and on the magnitude of the rise in ACTH levels and its absence in control animals with normal light-dark schedule. At the same time, a decrease in the magnitude of the rise in the level of ACTH preceded a decrease in the magnitude of the rise in plasma CORT (see **Figures 1** **-** **3**). Therefore, it can be considered that the inhibitory effect of CL on the secretion of CORT in young monkeys is due to the inhibition of ACTH secretion.

Apparently, the inhibitory effect of CL on stress activation of ACTH secretion in young animals is conditioned by the SCN-mediated inhibition of activation of AVP secretion from the hypothalamic PVN that controls the release of ACTH from the pituitary gland. This is indicated, on the one hand, by the literature data on the existence of such a neuronal pathway, through which information about the illumination of the environment is transmitted from the SCN to the adrenal cortex through the activation of CRH/AVP containing neurons of the medial parvocellular PVN and modulation of the circadian secretion of CRH and AVP, as well as pituitary ACTH and glucocorticoids by the adrenal cortex (24, 26, 28, 30). On the other hand, the inhibitory effect of CL on stress activation of ACTH secretion is confirmed by our results on the test with AVP, which revealed an inhibitory effect of CL on the magnitude of the rise in ACTH secretion and its absence in control animals with normal light-dark schedule (see **Figures 1** **-** **3**). CRH, apparently, does not play an important role in the mechanism of inhibition of HPA axis stress reactivity by CL, since the test with CRH not only did not reveal a decrease in the magnitude of the rise in ACTH levels in the behaviorally combined group of young animals (see **Table 2**), but even stimulated a significant elevated ACTH levels in animals with DAB (see **Figures 1** and **2**). It should be noted that earlier, under conditions of normal light-dark schedule we also noted a higher ACTH response to the test with CRH in young animals with DAB than in animals with SB (6). In addition, a higher rise in ACTH levels in response to CRH administration was characteristic of animals with DAВ compared with animals with SB in the basal period in our experiment (see **Figures 1** and **2**).

The absence of significant changes in the dynamics of the rise of the CORT level in response to the injection of CRH on the background of CL compared with the basal period in most of the studied time intervals, as well as in the area of the CORT response (see **Figures 1D** and **2B**) along with a significantly higher rise ACTH secretion in DAB animals in CL conditions (see **Figure 1C**) appears to be due to the inhibitory effect of CL on sensitivity of the adrenal cortex to ACTH. Indeed, a number of authors have shown that SCN, which receives light/dark information from the environment, has a direct effect on the regulation of glucociorticoid circadian rhythms mediated by neural projections into the hypothalamic PVN. PVN, in turn, switches the light signal to the HPA axis, providing a basis for diurnal fluctuations of glucocorticoids with their peak concentrations early in the morning and their nadir levels at night for humans and nonhuman primates (15, 25, 29, 30, 65). In addition, SCN appears to modulate the sensitivity of the adrenal cortex to ACTH in a time-of-day manner, possibly mediated by an extrahypophysial autonomic pathway using peripheral circadian clocks localized in the adrenal cortex (25, 32, 35).

Available literature data on the effect of CL on CRH are rare. But they indicate a mismatch in the rhythms of the concentration of CRH in the hypothalamus or CRH mRNA expression in the PVN, ACTH and corticosterone in the blood plasma (66, 67). Apparently, the classical scheme of regulation of the HPA axis function, in particular under stress exposure, is disturbed in conditions of CL, and the regulation of glucocorticoid secretion using the extrahypophyseal autonomous pathway becomes important, accompanied by a disorder of the sensitivity of adrenal corticocytes to ACTH, as well as the sensitivity of pituitary corticotrophs to AVP, which can lead to the altered response of the adrenals to stress exposure.

In old animals an inhibitory effect of CL on the magnitude of the CORT level rise in response to ASE was noted mainly in the absence of corresponding changes in the rise of plasma ACTH (see **Figures 5**, **6**). Apparently, in old animals, the inhibitory effect of CL on stress reactivity of the adrenal cortex is due to a decrease in the sensitivity of the adrenal cortex to ACTH, which is modulated by indirect extrahypophyseal autonomic pathway from the SCN to the adrenal cortex in a time-of-day manner (25, 30–35). This conclusion is also supported by the results of tests with CRH and AVP, which did not reveal statistically significant inhibitory effects of CL on the secretion of ACTH and CORT in old animals (**Figures 5** and **6**). In addition, in old animals under CL conditions, a more pronounced circadian decrease in the concentration of CORT at 15.00 was revealed than in the basal period, that is, the time of the onset of stress exposure (see **Figure 9**).

However, it should be noted that in some old animals under chronic CL there was a mild decrease in the magnitude of the rise in ACTH concentration in response to ASE and AVP administration along with a decrease in the magnitude of the rise in the CORT level (see **Results** for old monkeys). In this regard, it can be assumed that although in old animals CL inhibits mainly a sensitivity of the adrenal cortex to ACTH *via* the extrahypophyseal autonomic tract, in some individuals, a decrease in the secretion of CORT in response to ASE observed due to inhibition of ACTH secretion, that is, similar to that in young animals.

It should be noted that, although in the groups of young and old female rhesus monkeys, in response to the injection of CRH, there was no decrease in ACTH (and even an increase in young animals with DAB), and no decrease in the response area of ​​CORT and its levels in most of the studied intervals was observed, there was a statistically significant decrease in the concentration of CORT 15 min after the administration of CRH in all animals (see **Figures 1** and **5**). Most likely, exogenous CRH on the background of CL can briefly decrease the sensitivity of the adrenal glands to ACTH in young animals and the adrenals to ACTH or the splanchnic nerve in most old animals.

Since a number of studies assign MEL an important role in the regulation of circadian secretion of CORT (36, 37), and numerous data indicate that a light at night leads to a rapid and pronounced decrease in the secretion of MEL (12, 13, 21–23, 68, 69), we tried to evaluate the age-related and individual features of pineal MEL secretion under CL in our experiment. Therefore, when planning the experiment, we assumed that CL may be accompanied by a decrease in MEL secretion, and this phenomenon could be the main one in the supposed impairment of HPA axis stress reactivity. However, contrary to the expected decrease in the secretion of MEL under chronic CL, we have never revealed a significant decrease in the concentration of MEL at night in either young or old animals, regardless of the behavioral characteristics (see **Figures 4** and **9**). Therefore, we believe that the inhibitory effect of CL on HPA axis stress reactivity in our experiments is not due to a decrease in MEL.

Our finding that there were no significant changes in pineal MEL secretion under CL is consistent with a number of publications that also did not reveal a decrease in MEL secretion as a result of artificial light at night in humans and diurnal animal species (12, 13, 20, 70–72). In addition, a significant role was reported for the duration, brightness, features of the spectral characteristics of night lighting and the places of contact of illumination with the human and animal body (ocular or extraocular illumination) on the characteristics of pineal secretion of MEL (12, 20, 70). In particular, it was found that a decrease in the secretion of MEL under night lighting is characteristic only when the retina is exposed to blue, but not red light (13, 16, 70, 73, 74). The absence of an expected decrease in MEL secretion under night light, that we found, could be due to changes in the spectrum of polychromatic white light emitted by the discussed LED lamps (see **Methods**) compared to sunlight. Indeed, according to the characteristics of the LED lamps we use, provided by the distributor, the spectrum emitted by these lamps was characterized by 16 times less activity for short-wave blue light and only 5 times less activity for long-wave red light compared to the spectrum of natural sunlight. In addition, our use of a prolonged (several weeks) CL can neutralize the melatonin-inhibiting effect of green light, as long-term green light illumination has been shown to rapidly desensitize melatonin suppression (75).

It should be noted that, despite the absence of changes in the concentration of MEL in young and old female rhesus monkeys at different times of the day (09.00, 15.00, 22.00) under CL, we found a significant decrease in the concentration of CORT on the background of CL in all animals in 15.00, as well as its increase in young animals at 22.00. In addition, in young animals there was a statistically significant decrease in the amplitude of the circadian rhythm of CORT (the difference between its concentration at 09.00h and 22.00h) (**Figures 4** and **9**). These data, as well as the above data on a decrease in the stress reactivity of the HPA axis under CL, indicate the damaging effect of CL on the function of the HPA axis.

The identified age-related differences in the function of the HPA axis under CL may be dependent on the age-related changes in the axis “retina – SCN - tissue clock genes”, which were noted by various authors (20, 76–78). In particular, age-related changes have been described for the biochemistry and morphology of SCN (20, 79), the expression of a number of biologically active peptides of SCN (vasoactive intestinal polypeptide; AVP) (80, 81) and an age-related decrease in the amplitude of circadian rhythms of electrical activity in SCN (82). According to some authors, both constriction cause progressive age-related losses in circadian photoreception in terms of phase shifts and melatonin suppression (76, 83).

The revealed phenomenon of the inhibitory effect of CL on the stress reactivity of HPA axis may be of significant practical importance. On the one hand, the findings suggest that chronic CL with LED lamps designed for residential, office and commercial environments may impairs the response of the HPA axis to stress exposure. This finding, apparently, can be used to correct increased vulnerability to stress in individuals with preexisting anxiety and depression stress behaviors. Thus, it is known that increased stress reactivity of the HPA axis often detected in persons with an anxiety and depression-prone behavior, as well as patients with some types of clinically diagnosed depression (6, 60, 62, 63). Perhaps, short-term lighting courses in the evening or at night would be effective to correct increased vulnerability the HPA axis to acute stress. Currently, light therapy at night already is carried out for the treatment of psychiatric diseases, in particular depression (15, 84, 85), sleep disorders (15, 86), neurodegenerative diseases (87, 88). Perhaps it is based, at least in part, on the inhibitory effect of light on the stress reactivity of the HPA axis that we have identified. On the other hand, the CL induced decrease in stress reactivity of the adrenals in behaviorally normal animals suggests a potential risk of reducing the adaptive capacity of the organism under continuous CL conditions. Thus, a decrease in adaptive abilities was noted among residents of the Far North and members of expeditions in Antarctica, as well as among people who work the night shift for a long time (89–94).

## Conclusion

Thus, experiments on the study of the features of the HPA axis response to acute stress under conditions of chronic round-the-clock illumination (CL) on the model of young adult and old female rhesus monkeys revealed a pronounced decrease in the magnitude of the rise in the CORT level in response to acute stress exposure (ASE), the mechanisms of which were age-dependent. Young animals were characterized by an inhibitory effect of CL on the adrenal cortex function due to inhibition of the pituitary ACTH secretion. In turn, the inhibitory effect of CL on ACTH secretion seems to be due to the inhibition of the ACTH-stimulating effects of vasopressin. At the same time, in the majority of old animals, an inhibitory effect of CL directly on the function of the adrenal cortex without the participation of the adenohypophysis were revealed. The inhibitory effect of CL on the function of the adrenal cortex was detected in all animals, regardless of behavior, although a number of individual differences were also noted, apparently not of a fundamental nature for the revealed phenomenon. The observed CL inhibition of adrenal cortical reactivity to ASE may be useful to correct increased vulnerability to ASE observed in individuals with preexisting anxiety and depression-like stress behaviors. On the other hand, the CL induced decrease in adrenal stress reactivity of behaviorally normal animals suggests a potential risk of reducing the adaptive capacity of the organism under conditions of continuous light exposure.

The conclusion made about the inhibitory effect of CL on the stress reactivity of the adrenal cortex in old animals has some limitations, since in the experiment with ASE there was no control group, and the comparison of the stress reactivity of the HPA axis in CL conditions was carried out relative to the stress reactivity of the HPA axis in these same animals in the basal period.

## Data availability statement

The datasets presented in this study can be found in online repositories. The names of the repository/repositories and accession number(s) can be found in the article/**Supplementary Material**.

## Ethics statement

The animal study was reviewed and approved by the Ethics Committee of the Research Institute of Medical Primatology, Sochi, Russia.

## Author contributions

NG: study design, supervision of performing experiments, analysis of research results, writing the manuscript; OC: performing experiments, statistical analysis of results, preparation of tables and figures; TO: performing experiments, measurement of hormone levels. All authors have approved the final version of manuscript.
